# Genetic Variation in the *blaZ* Gene Leading to the BORSA Phenotype in *Staphylococcus aureus*

**DOI:** 10.3390/antibiotics14050449

**Published:** 2025-04-29

**Authors:** Mia Aarris, Frederik Boëtius Hertz, Karen Leth Nielsen, Alexander Sato, Helle Krogh Johansen, Henrik Westh, Michael Kemp, Svend Ellermann-Eriksen, Anders Løbner-Olesen, Niels Frimodt-Møller, Godefroid Charbon

**Affiliations:** 1 Department of Clinical Microbiology, Rigshospitalet, 2100 Copenhagen, Denmark; miai@ssi.dk (M.A.); frederik.boetius.hertz@regionh.dk (F.B.H.); niels.frimodt-moeller@regionh.dk (N.F.-M.); 2 Department of Immunology & Microbiology, University of Copenhagen, 2200 Copenhagen, Denmark; 3 Institute of Biology, University of Copenhagen, 2200 Copenhagen, Denmarklobner@bio.ku.dk (A.L.-O.); 4 Department of Clinical Medicine, Faculty of Health and Medical Sciences, University of Copenhagen, 2100 Copenhagen, Denmark; mkemp@regionsjaelland.dk; 5 Department of Clinical Microbiology, Hvidovre Hospital, 2650 Hvidovre, Denmark; 6 Department of Clinical Microbiology, Odense University Hospital, 5000 Odense, Denmark; 7 Department of Clinical Microbiology, Aarhus University Hospital, 8200 Aarhus, Denmark

**Keywords:** BlaZ, methicillin-susceptible *S. aureus* (MSSA), borderline oxacillin-resistant (BORSA), oxacillin

## Abstract

Background/Objectives: *Staphylococcus aureus* is a leading cause of bacteraemia in Danish hospitals. Approximately 70% of clinical *S. aureus* isolates are penicillin-resistant, which is predominantly due to *blaZ*-mediated β-lactamase production. Methods: A collection of 489 *S. aureus* strains derived from bacteraemia were cultured and their genomes sequenced. Results: From this collection, 71% of isolates were methicillin-susceptible *S. aureus* (MSSA) harbouring *blaZ*. While most isolates contained the *blaZ* gene belonging to the well-characterised A, B, C and D variants, three strains (1%) produced a BlaZ protein characterised by having threonine residues on both positions 128 and 216 and, therefore, belonged to neither of the established *blaZ* variants. We named this variant, variant F. We report that clinical isolates expressing *blaZ* variant F were resistant to oxacillin. The β-lactamase production phenotype in isolates carrying either of the A, B, C or D variants was only weakly discernible on MIC gradient strip and disk diffusion tests. When the β-lactamases were expressed either from a T7 promoter or from their endogenous promoters in *Escherichia coli*, variant F was significantly better at degrading ampicillin than variant A. We also showed that variant F conferred oxacillin resistance when expressed in an isogenic *S. aureus* strain, while variant A did not. Finally, we demonstrated that the F variant threonine 216 played a role in the enzyme’s superior activity. Conclusions: Our findings demonstrate that the new F variant of BlaZ is sufficient to render *S. aureus* a BORSA strain, which is superior in the degradation of common anti-staphylococcal β-lactam antibiotics, such as benzylpenicillin, cloxacillin, and oxacillin. It is sensitive to β-lactamase inhibitors and rapidly degrades nitrocefin. We provide a genetic explanation for the borderline oxacillin-resistant *S. aureus* (BORSA) phenotype.

## 1. Introduction

*S. aureus* is a leading cause of bloodstream infections, which can result in life-threatening conditions, including endocarditis or septic shock. In Denmark, the incidence of *S. aureus* bacteremia increased by 76% from 2014 to 2023, with 1.5% of cases attributed to methicillin-resistant *S. aureus* (MRSA). While MRSA remains a clinical concern, methicillin-susceptible strains (MSSA), and even penicillin-susceptible PSSA, are now on the rise globally [1]. The mortality associated with MSSA bacteraemia is about 10%, stressing the importance of understanding the resistance profile of these bacteria.

In isolates that are not MRSA (*mecA*/*C* negative, cefoxitin MIC ≤ 2 mg/L), penicillin resistance is primarily driven by *blaZ*-mediated β-lactamase production. Expression of *blaZ* is induced when β-lactams are present in the environment. The regulation is controlled by two genes, *blaI* (repressor) and *blaR1* (sensor), that are located in a gene cassette, with *blaZ* often carried on a plasmid [2,3,4].

The β-lactamase encoded by *blaZ* belongs to the β-lactamase subgroup 2a within molecular class A [5]. Five variants of the *blaZ* gene (A, B, C, D and E) have been characterised in *S. aureus* (Table 1) and can be distinguished based on the amino acid residues on positions 128 and 216 according to Ambler’s standard numbering scheme [6]. These residues are kinetically important for β-lactam degradation and, consequently, the β-lactam susceptibility of *S. aureus* [7,8]. The phenotypic resistance profile of MSSA is heterogeneous. MSSAs with borderline oxacillin resistance (BORSA), characterised by oxacillin MICs of 1–8 mg/L, are of particular concern. This resistance not only poses a risk of treatment failure but can also result in the misidentification of MSSA as MRSA. BORSA strains, which do not carry *mecA*/*C*, remain susceptible to cefoxitin. The prevalence of BORSA strains has been estimated to be 5% of *S. aureus* isolates [9]. The genetic basis of BORSA resistance is still being studied and has been linked to the ‘hyperproduction’ of β-lactamase [10,11] (increased β-lactamase activity measured by nitrocefin assay), differential expression or mutation of genes encoding penicillin-binding proteins [12,13,14,15], and mutations in the *gdpP* regulator [16,17]. Isolates with the BORSA phenotype that are not directly dependent on *blaZ* are also referred as MODSA (modified methicillin-resistant *Staphylococcus aureus*) in the literature. To complicate matters, the phenotypic testing of MSSA is known to be difficult to interpret, with the results varying according to the test, the media used, the inoculum size, or by the subculturing of the original isolates [18,19,20,21]. The golden standards for penicillin resistance identification is PCR amplification of *blaZ* and the clover leaf assay [22].

In this study, we examined the distribution of *blaZ* variants in a collection of clinical *S. aureus* bloodstream isolates from three Danish university hospitals and compared their susceptibilities to various β-lactams using agar disk diffusion and MIC gradient strips. We identified a novel *blaZ* variant F, whose expression is sufficient to confer a BORSA phenotype. Strains carrying *blaZ* variant F are efficient at degrading nitrocefin, have an oxacillin MIC of 1–4 mg/L, a penicillin resistance that can be counteracted by β-lactamase inhibitors, and they are cefoxitin susceptible.

## 2. Results

### 2.1. Distribution of blaZ Variants in Clinical S. aureus Isolates

**Figure 1:**
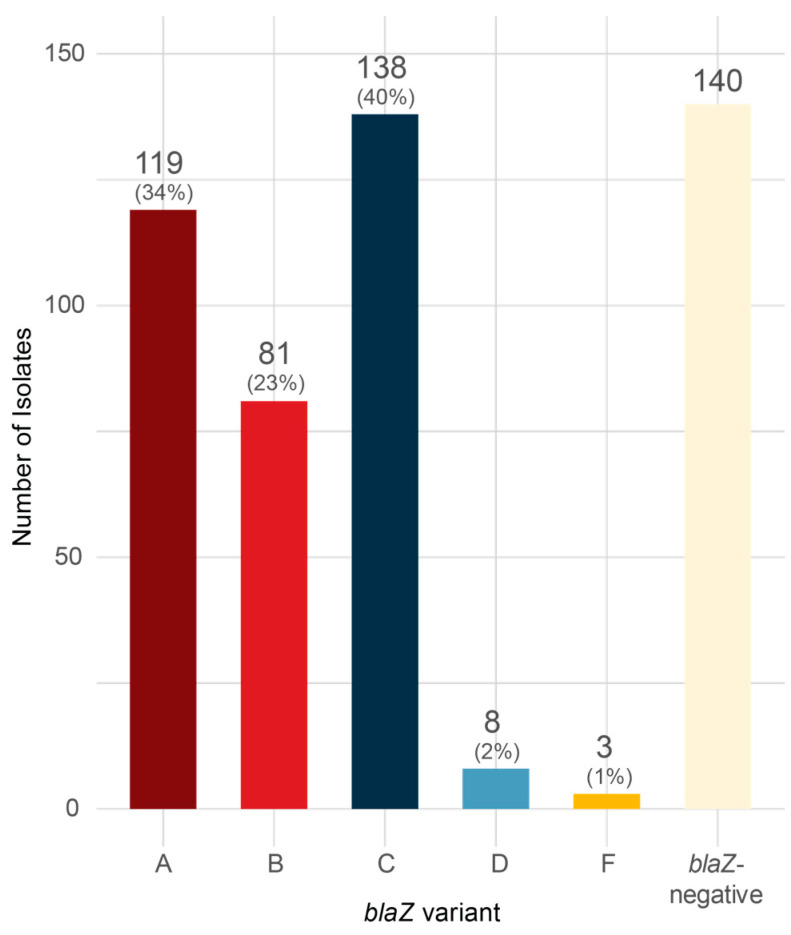
Distribution of *blaZ*-mediated β-lactamase variants in clinical *S. aureus* (*n* = 489) isolated from bacteraemia in three Danish University hospitals in 2019. The variants identified included the four well-described *blaZ* variants, A, B, C and D as well as a new variant F. No E variants were detected. The proportion of each variant among *blaZ*-carrying bacteria is indicated in percent.

We examined the distribution of *blaZ* variants in the genome sequence of clinical MSSA blood isolates (*mecA*/*C* absent) collected from three Danish university hospitals in 2019 and 2020. A total of 489 isolates were included in the study, of which 349 (71%) harboured *blaZ*. The majority of these isolates (99%) belonged to the well-described β-lactamase variants A–D, 34% variant A, 23% variant B, 40% variant C and 2% variant D, while no isolates carrying variant E [23] were detected (Figure 1).

Three isolates (1%) differed from the five previously described β-lactamase types by harbouring threonine residues on both position 128 and 216 in the protein sequence according to Ambler nomenclature [5,6] and were designated as the novel variant F. These bacteria were isolated from Aarhus University Hospital (AUH2145 and AUH2165) and Amager-Hvidovre Hospital (HVH359). The *blaIRZ* gene cassette was 100% identical in all three strains. The AUH isolates were genetically identical (MLST 188, CC1) and belonged to the same patient, while the HVH359 strain belonged to ST1 within CC1. All three carried *blaZ* on plasmids: AUH2145 and AUH2165 strains on a RepA_N/rep20 plasmid type and the HVH359 strain on a rep3/5a plasmid type 99.99% identical to pMW2 [24]. pMW2 that carries *blaZ* variant C originated from MRSA strain MW2.

### 2.2. Antibiotic Susceptibility of Clinical Isolates Carrying Variants A, B, C, D and F

To evaluate phenotypic differences in β-lactam resistance, antimicrobial susceptibility testing was performed on randomly selected *S. aureus* isolates harbouring variants A (*n* = 10), B (*n* = 10), or C (*n* = 10) and all isolates carrying variants D (*n* = 8) and F (*n* = 3). These isolates were tested against a range of clinically relevant anti-staphylococcal β-lactam antibiotics using agar disk diffusion and MIC gradient strips, with both methods generally showing a similar pattern of resistance (Table 2, Tables S1 and S2). The ATCC^®^ reference strain 29213, which produces BlaZ variant A, and nine strains (S) selected from the penicillin-susceptible *S. aureus* isolates (*blaZ* absent) were included in the study.

As expected for MSSAs, all isolates were found to be susceptible to cefoxitin (Table S2). All isolates carrying *blaZ* A–D were found to be less susceptible to the penicillins, benzylpenicillin, ampicillin, amoxicillin, and piperacillin (Table 2, Tables S1 and S2) than *blaZ*-lacking strains, although the susceptibility was only marginally decreased for most isolates. This is in line with the known difficulty to observe β-lactam resistance by classic phenotypic tests for *S. aureus*. The use of β-lactamase inhibitors weakly sensitised *blaZ* A–D-carrying isolates (Table 2, Tables S1 and S2).

However, two isolates carrying variant F were found to be significantly more resistant to benzylpenicillin and piperacillin than all other isolates, with MICs ranging from 1 to 32 mg/L and 3 to 48 mg/L, respectively (Table 2). Importantly, isolates carrying *blaZ* variant F were found to be susceptible to piperacillin when used in combination with tazobactam, which reduced the MIC from 12 mg/L to MIC 1 mg/L.

For most *blaZ*-carrying isolates, the susceptibility towards isoxazoyl-penicillins (oxacillin and cloxacillin) and the cephalosporin cefuroxime was somewhat similar to isolates that did not carry *blaZ* (Tables S1 and S2). Again, all isolates carrying *blaZ* variant F distinguished themselves by being clearly less susceptible to oxacillin and cloxacillin with MICs ranging from 1.5 to 4 mg/L and 0.5 to 1.5 mg/L, respectively (Table 2). Thus, strains with variant F possessed the hallmarks of a BORSA strain. As part of antibiotic susceptibility testing, we also tested the susceptibility towards meropenem and mecillinam for each *blaZ* variant without finding any significant difference (Table S2).

### 2.3. Comparison of Variants A, B, C, D and F in Escherichia coli

We hypothesised that the genetic variation in *blaZ* causes the β-lactam resistance observed in isolates carrying variant F.

To test this, a representative of each *blaZ* variant and *blaZ* variant A carried by reference strain ATCC^®^ 29213 were cloned into plasmid pET26b(+) and transformed into *E. coli*. Expression of *blaZ* from the T7 promoter was induced with 1 µM IPTG; subsequently, the antibiotic susceptibility of BlaZ-expressing *E. coli* cells was tested against benzylpenicillin, ampicillin, oxacillin, cloxacillin and cefuroxime (Table S3).

All MICs for *E. coli* were considerably higher than the MICs for *S. aureus*, as expected for the Gram-negative bacterium. *E. coli* cells expressing the β-lactamase variants showed similar susceptibility to all drugs with the exception of ampicillin. *E. coli* cells expressing variant F had a higher MIC (512 mg/L) than any other BlaZ variant (64–32 mg/L). No difference in MICs between the BlaZ variants was observed for oxacillin and cloxacillin, as *E. coli* is naturally resistant to these compounds.

**Figure 2:**
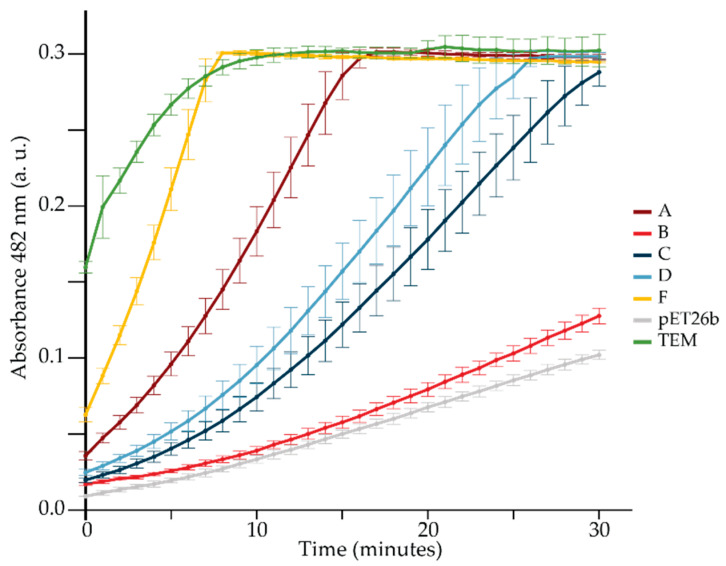
Nitrocefin hydrolysis by crude extracts of *E. coli* expressing *blaZ* variants. Equal amounts of crude *E. coli* extracts expressing *blaZ* variants (A, B, C, D, F) and a TEM-1 β-lactamase from the pACYC177 plasmid (positive control) were incubated at 37 °C in the presence of nitrocefin. The β-lactamase activity was monitored by measuring the absorbance at 482 nm over time. An extract of *E. coli* carrying the pET26b vector served as a negative control.

The β-lactamase enzymatic activity was then estimated using the chromogenic cephalosporin nitrocefin as a substrate and extracts of *E. coli* cells producing each of the staphylococcal BlaZ variants (Figure 2).

Of the BlaZ variants, variant F was clearly the most efficient at degrading nitrocefin, followed by BlaZ ATCC 29213 (a variant A), while variant B was the least efficient. Fast degradation of nitrocefin in the original assay defined a BORSA strain as a BlaZ ‘hyperproducer’ [10,11].

The entire *blaI-blaR-blaZ* cassette from the *S. aureus* isolate carrying variant A (HVH341) and *S. aureus* isolate carrying variant F (AUH2165) were cloned into a synthetic plasmid with the p15A origin of replication (for *E. coli*) and the RepA_N origin of replication of pSK41 (for *S. aureus*) [25]. Because we hypothesised that threonine 216 plays a role in the enhanced resistance phenotype of cells expressing variant F, we mutated Serine 216 of variant A to threonine, i.e., create variant F.

*E. coli* cells carrying the plasmid pBL_F with the *blaIRZ* cassette of variant F showed significantly higher resistance to ampicillin (MIC > 512 mg/L) than cells carrying the regulon of variant A on pBL_A (MIC of 16 mg/L) (Table S4). The serine to threonine substitution introduced into variant A BlaZ on pBL_AF improved the resistance phenotype (MIC of 128 mg/L) but not to the same extent as variant F (MIC > 512 mg/L), indicating that other mutations present in the sequence of variant F also contribute to its resistance phenotype in *E. coli* (Figure S1).

### 2.4. Antibiotic Resistance of Variants A and F in the S. aureus Newman Strain

The *S. aureus* Newman strain [26] was transformed with synthetic plasmids carrying *blaIRZ*. When tested by broth dilution, the Newman strain carrying pBL_F was significantly less sensitive to benzylpenicillin, oxacillin, and cloxacillin than the Newman strain carrying pBL_A (Table 3). The strain carrying *blaZ* type A exhibited a weak resistance phenotype to benzylpenicillin compared to the Newman strain carrying a control plasmid pBL_N, which is devoid of the *blaIRZ* cassette. However, the susceptibility to cloxacillin and oxacillin was marginally improved in a strain carrying pBL_A.

**Figure 3:**
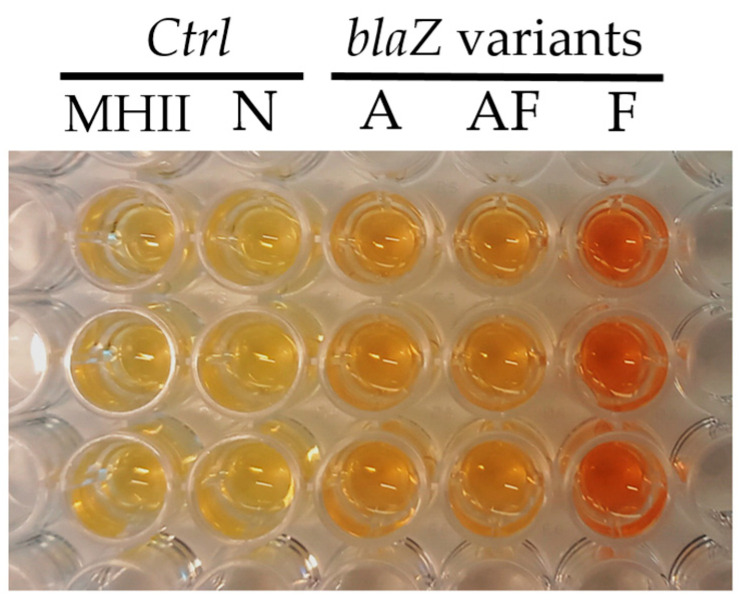
Nitrocefin assay of *S. aureus* Newman strains carrying *blaIRZ* gene cassettes. Strains carrying pBL_A, pBL_AF and pBL_F or pBL_N backbone vectors were grown for 16 h at 37 °C and diluted to an OD_600_ of 0.1. The cultures were incubated at 37 °C for 30 min in the presence of 0.5 μg/mL nitrocefin. MHII medium with nitrocefin was used as control.

The serine to threonine mutation carried by pBL_AF only slightly increased the MIC, again indicating that other mutations must contribute to the phenotype of variant F. We note however, that in the case of benzylpenicillin, the resistance phenotype of strains carrying variant F was much less pronounced when tested with MIC gradient strips (Table 3). As expected, the strains carrying pBL_F were clearly more efficient at degrading nitrocefin (Figure 3).

## 3. Discussion

Among 489 MSSA blood isolates collected from three Danish university hospitals, 349 strains were found to carry *blaZ*, with 97% of these strains belonging to Ambler types A (34%), B (23%) and C (40%) and only 2% to type D. A very similar distribution of variants has been reported elsewhere [20,27]. We report here the identification of three isolates carrying a new Ambler type that we named F due to the presence of threonines at positions 128 and 216 of BlaZ. Isolates carrying *blaZ* A–D were found to be generally slightly less susceptible to penicillins than strains that did not carry *blaZ*. These isolates were as susceptible to isoxazoyl-penicillins and cephalosporins as isolates that did not carry *blaZ*.

However, the presence of the *blaZ* variant F results in a significant decrease in susceptibility to benzylpenicillin and piperacillin. Importantly, the use of tazobactam counteracts the activity of the *blaZ* F variant. Isolates carrying variant F were also less susceptible to the isoxazolyl-penicillins oxacillin and cloxacillin while remaining susceptible to cefuroxime. *E. coli* cells expressing BlaZ variant F were consistently better at degrading nitrocefin and were less susceptible to ampicillin than cells expressing any other BlaZ type.

When the *blaIRZ* cassette was cloned and expressed in an isogenic *S. aureus* strain, strains carrying variant F became resistant to benzylpenicillin and were significantly less susceptible to oxacillin than strains expressing variant A, while susceptibility to cloxacillin was slightly decreased. Strains expressing variant A were as susceptible to oxacillin and cloxacillin as *blaZ*-negative isolates.

Because serine at position 216 is reported to be important for the catalytic activity of β-lactamase, we mutated serine 216 in variant A (Thr 128/Ser 216) into threonine, aiming to mimic the activity of the new variant F (Thr 128/Thr 216). However, this only slightly enhanced the resistance phenotype, indicating that variant F is defined by more than just the presence of threonine 128 and threonine 216. In fact, variant F differs from other variants at additional positions, such as in the omega loop (Figure S1), which is known to influence both the spectrum and efficiency of β-lactamases [28].

So far, by searching for BlaZ protein that possess threonine 128 and threonine 216 in NCBI databases, we found few additional *S. aureus* strains carrying variant F. We found the variant in strains originating from Denmark, the UK, the USA, the Netherlands and Mexico, among other countries (Table S5, Figure S2). *S. aureus* strains carrying *BlaZ* F are not limited to MSSA, as the variant was also found in MRSAs, such as an isolate from Hvidovre hospital in Denmark (strain M8420). We found only one example (CP170411.1) that was 100% identical at the DNA level to variant F carried by HVH359, AUH2145 and AUH2165. This is surprising, especially considering the fact that it requires only the mutation of one single base in variants A and C to become variant F. We also identified *blaZ* variant F in multiple coagulase-negative staphylococci, such as *S. haemolyticus* (CP035541.1) and *S. epidermidis* (CP064549.1) (100% sequence identity with BlaZ F variant DNA carried by HVH359, AUH2145 and AUH2165).

Nevertheless, we describe here that a variation in *blaZ* is sufficient to confer the BORSA phenotype, providing an explanation for the β-lactamase ‘hyperproduction’ phenotype [10,11]. Although *blaZ* variants A and C have been reported in clinical BORSA strains [29], it is not clear if variation in *blaZ* also contributes to their resistance phenotype. Thus, exhaustive mapping of all BlaZ variation conferring oxacillin resistance would be instrumental to predict the BORSA phenotype.

One limitation of our study is that the *blaIRZ* gene cassette was 100% identical in all three isolates harbouring *blaZ* variant F. Characterising other isolates carrying variant F that diverge in sequence may provide additional insights into the significance of threonine at positions 128 and 216.

In conclusion, a new *blaZ*-mediated β-lactamase, F, has been discovered in clinical methicillin-susceptible and penicillin-resistant *S. aureus* that was superior in the degradation of common anti-staphylococcal β-lactam antibiotics, such as benzylpenicillin and oxacillin/cloxacillin compared to four previously characterised *blaZ* variants (A, B, C, D). While possible problems with treatment failure of these penicillins for the new variant have not been observed at present, screening with oxacillin for detecting MRSA could lead to misclassification of variant F.

## 4. Materials and Methods

### 4.1. Isolate Collection

Clinical *S. aureus* isolates (*n* = 489) were isolated from positive blood cultures from three Danish University hospitals, Amager-Hvidovre Hospital (HVH), Aarhus University Hospital (AUH) and Odense University Hospital (OUH), in 2019 and 2020 (Table S6).

### 4.2. Whole Genome Sequencing & Bioinformatics

Bacterial DNA was purified with a Blood and Tissue Kit (Qiagen). The isolates were whole genome sequenced using Nextera XT prepared libraries (2 × 150 bp, Illumina). The isolates were typed with MLST v2.0, applying the PubMLST database. Assemblies were generated with Shovill using SPAdes v3.14. Resistance genes were identified with the Resfinder v 4.2.3 database using Abricate. The genomes were annotated with Prokka v. 1.14.0.

The assembled genomes were analysed in Geneious Prime 2021.2.2 using the BLASTN tool to compare them against a *blaZ* reference sequence from USA300 (GenBank accession no. NG_055999). Subsequently, manual examination was conducted to identify all nonsynonymous mutations.

### 4.3. Antibiotic Susceptibility Testing

The methicillin-susceptible *S. aureus* (MSSA) isolates were tested against a range of antibiotics using agar disk diffusion and MIC gradient strips according to EUCAST guidelines. The bacteria were tested with 11 different antibiotic disks (Oxoid, Thermo Fischer, Basingstoke, UK): penicillin G (1 unit), ampicillin (2 μg), amoxicillin+clavulanic acid (30 μg (2:1)), ampicillin+sulbactam (20 μg (1:1)), oxacillin (1 μg), piperacillin (100 μg), piperacillin-tazobactam (110 μg (10:1)), mecillinam (25 μg), cefoxitin (30 μg), cefuroxime (30 μg) and meropenem (10 μg). The disks were applied with an Oxoid^TM^ (Basingstoke, UK) antimicrobial susceptibility disc dispenser. The minimum inhibitory concentration (MIC) was determined with MIC gradient strips (Liofilchem^®^, Roseto degli Abruzzi, Italy) containing penicillin, piperacillin, amoxicillin, oxacillin, cloxacillin, amoxicillin-clavulanic acid, ampicillin-sulbactam, piperacillin-tazobactam, cefuroxime. Disk diffusion was performed in biological duplicates, and MICs using MIC gradient strips were executed in biological singlicate.

The clover leaf test was performed on a 5% sheep blood agar plate (SSI Diagnostica A/S, Copenhagen, Denmark) using a 10 unit benzylpenicillin tablet (Rosco, Albertslund, Denmark). The plate was pre-seeded with *Micrococcus luteus* (*Sarcina lutea*), which was matched to a 0.5 McFarland turbidity standard in 0.9% NaCl. The isolates were streaked out in a cross on the *Micrococcus*-seeded plate, and the benzylpenicillin tablet (Rosco) was placed in the centre of the cross with sterile forceps. The plates were incubated for 18–24 h at 35–37 °C. The plates were read by visual examination compared to a negative and positive control.

Susceptibility testing of *blaZ*-encoded β-lactamase-producing *E. coli* strains was done using broth microdilution. The broth microdilution was performed according to the EUCAST guidelines. *blaZ* expression was induced by adding 1 µM IPTG to the growth media. Susceptibility testing was executed in biological triplicate.

### 4.4. Cloning of the blaZ in pET26b(+) Plasmid

DNA of selected clinical staphylococcal isolates was purified as follows: 2 mL overnight cultures (ON) were centrifuged at 8000 rpm for 15 min at 10 °C, and the pellet was washed twice with 0.9% NaCl. The pellet was resuspended in 200 μL of 10 mM Tris-HCl (pH 8), 50 μL lysozyme (10 mg/mL) and lysostaphin to a final concentration of 250 μg/mL and incubated at 37 °C for 1 h with occasional mixing. After incubation, 500 μL of lysis buffer (50 mM Tris, 100 mM EDTA, 1% SDS, pH 8), 1 mg/mL proteinase-K and 100 μg/mL RNase A was added, and the sample was incubated at 56 °C for 1 h. After incubation, 500 μL of phenol:chloroform:isoamyl alcohol 25:24:1 (PCIA) was added. The sample was vortexed for 1 min then centrifuged at 12,000 rpm for 10 min, and the upper layer was transferred to a new tube. This PCIA step was repeated. Subsequently, 500 μL chloroform:isoamyl alcohol (24:1) was added, and the sample was centrifuged at 12,000 rpm for 5 min. The upper layer was transferred to a new tube, and this step was repeated. Then, 25 μL of 5 M NaCl, 1 μL GlycoBlue™ Coprecipitant (15 mg/mL, Invitrogen, Vilnius, Lithuania) and 1 mL of 96% ethanol were added to the supernatant, and the sample was kept on ice for 20 min. The sample was centrifuged at 16,000 rpm at 4 °C for 20 min, and the supernatant was discarded. The pellet was washed in 70% ethanol without resuspension and centrifuged at 10,000 rpm at 4 °C for 10 min. The pellet was left to dry until all ethanol evaporated. Finally, the pellet was resuspended in 200 μL 10 mM Tris pH 8, and 0.1 mM EDTA buffer.

*blaZ* was PCR amplified using the purified *S. aureus* DNA as template and the following primers (Table S7):

Forward primer #1 was used to clone variants A and D. Forward primer #2 was used for variant B. Forward primer #3 was used for variant C. Forward primer #4 was used for variant F. Reverse primer #5 was used for variants A, C, D, ATCC and F. Reverse primer #6 was used for variant B.

PCR products were digested with the restriction enzymes XhoI and BcuI, ligated with pET26b(+), and digested with XhoI and XbaI. The plasmid constructs were sequence verified and transformed into *E. coli* MG1655 (λDE3) lab strain ALO6511.

### 4.5. Cloning of the blaIRZ Cassette in S. aureus Plasmid

*blaIRZ* cassette from variant A and from variant F were PCR amplified using the purified *S. aureus* DNA as template and the following primers: primer #7 and primer #8 for variant A, and primer #9 and primer #10 for variant F. The PCR products were cloned into pACYC184 cut with EcoRV.

The pSK41 plasmid origin of replication of *S. aureus* was amplified using pSK9067 [25], primer #11 and primer #12. The PCR product was cut with Eco52I and BamHI and cloned into pACYC184 *blaI-blaR-blaZ* cut with Eco52I and BamHI to create pSATO_A and pSATO_F. The erythromycin resistance gene from pSK9067 was amplified using primer #13 and primer #14. The PCR product was cut with EcoRI and cloned into pACYC184 cut with EcoRI to create pSATO2.

To create synthetic vectors carrying *blaI-blaR-blaZ* regulons, 4 PCR amplicons were assembled using GeneArt™ Gibson Assembly^®^ HiFi Master Mix (Thermo Fisher, Vilnius, Lithuania).

We used pSATO_A, pSATO_F and pACYC184 as templates and primer #15 and primer #16 to create *blaI-blaR-blaZ* regulons A and F and the negative control fragment. We used pSATO_A and pSATO_F as templates and primers: primer #17 and primer #18 to create the Ori fragment. We used pACYC184 as the template and primer #19 and primer #20 to create the CmR fragment. We used pSATO_2 as the template and primer #21 and primer #22 to create the EryR fragment. The four fragments were assembled to create pBL_A, pBL_F and pBL_N. To introduce a point mutation in the *blaIRZ* cassette, two fragments were PCR amplified using pBL_A as the template and primer #15 and primer #23 or primer #16 and primer #24. The two fragments were assembled with CmR, EryR and Ori fragments using GeneArt™ Gibson Assembly^®^ HiFi Master Mix to create pBL_AF.

Plasmids pBL_A, pBLAF, pBL_F and pBL_N were transformed into *E. coli* IMB08 before transformation in the *S. aureus* Newman strain using the procedure described in [30]. Transformants were selected on TSB broth supplemented with 20 mg/mL erythromycin.

### 4.6. Nitrocefin Assay on Cell Extracts

The nitrocefin assay was performed according to [31]. The assay was performed with raw *E. coli* lysate prepared as follows: ON cultures were diluted 100-fold in LB medium containing 50 μg/mL kanamycin, grown to OD_600_ = 0.3 and *blaZ* expression was induced by adding 1 mM isopropyl β-D-1-thiogalactopyranoside (IPTG) for 3 h. The cells were harvested by centrifugation and sonicated for 20 cycles (30 s of sonication, 30 s cooling) at 4 °C using a Bioruptor^®^ (Seraing, Belgium) Plus sonication device. The protein concentration was determined with Bradford reagent (Thermo Fischer), and the samples were diluted in phosphate buffer (PBS) to standardise the β-lactamase contents. Then, 10 µL standardised *E. coli* lysate was added to 90 μL (50 μg/mL) nitrocefin stock (Sigma-Aldrich). The degradation of nitrocefin was monitored by measuring absorbance at 482 nm in the BioTek Synergy H1 microplate reader for 30 min at 37 °C. The assay was performed in triplicate.

## Figures and Tables

**Table 1:** *blaZ* variants in *S. aureus* according to Ambler’s standard numbering scheme.

*blaZ* Variants	Amino Acid 128	Amino Acid 216
A	Thr	Ser
B	Lys	Asn
C	Thr	Asn
D	Ala	Ser
E	Leu	Ser
F	Thr	Thr

**Table 2:** Antimicrobial susceptibility testing of clinical *S. aureus* isolates using MIC gradient strips. Values displayed are the MIC median (mg/L) and ranges for variants A, B and C (*n* = 10), variant D (*n* = 8), and variant F (*n*= 3).

Antibiotic	*blaZ* variants					Controls	
	A	B	C	D	F	S	ATCC29213
Benzylpenicillin	0.22 (0.094–0.38)	0.19 (0.125–0.75)	0.22 (0.125–0.38)	0.125 (0.094–0.25)	4 (1–32)	0.064 (0.064)	0.19 (0.19)
Piperacillin	1 (1–2)	2 (1.5–4)	3 (1.5–4)	2 (0.5–4)	12 (3–48)	0.38 (0.38)	1.5 (1.5)
Piperacillin-tazobactam	1 (0.5–1.5)	2 (0.75–3)	1.5 (0.75–1.5)	1 (0.75–2)	1 (0.75–2)	0.38 (0.38)	0.75 (0.75)
Amoxicillin	0.32 (0.19–0.75)	0.75 (0.38–1.5)	0.5 (0.38–1)	0.5 (0.125–0.75)	1 (0.75–1.5)	0.125 (0.125)	0.38 (0.38)
Oxacillin	0.25 (0.125–0.38)	0.38 (0.25–0.5)	0.38 (0.25–0.75)	0.32 (0.25–0.5)	1.5 (1.5–4)	0.125 (0.125)	0.19 (0.19)
Cloxacillin	0.125 (0.094–0.19)	0.19 (0.125–0.25)	0.19 (0.19–0.38)	0.19 (0.125–0.25)	0.75 (0.5–1.5)	0.125 (0.125)	0.19 (0.19)

**Table 3:** MIC for *S. aureus* Newman strains carrying *blaIRZ* gene cassettes. Antibiotic susceptibility tests (ASTs) for benzylpenicillin, oxacillin and cloxacillin were determined using broth microdilution in MHII (BMD) and MIC gradient strips (E-test). The *blaIRZ* gene cassette from variant A, variant A carrying mutation S216T (A–F) and variant F are carried on plasmids pBL_A, pBL_AF and pBL_F, respectively. The pBL_N backbone vector does not carry the *blaIRZ* cassette. ATCC 25923 (*blaZ* negative) was used as a negative control. Values displayed are the MIC (mg/L) median and ranges for BMD (*n* = 3) and MIC gradient strips (*n* = 4).

Antibiotic	AST Method	*blaZ* Variants			Controls	
		A	A–F	F	N	ATCC25923
Benzylpenicillin	BMD	1 (1)	2 (2)	32 (32)	<0.03 (<0.03)	0.0625 (<0.03–0.0625)
	E-test	0.38 (0.38)	0.5 (0.38–0.5)	1.5 (1.5–3)	0.32 (0.32)	-
Oxacillin	BMD	0.25 (0.25)	0.5 (0.5)	2 (2–4)	0.125 (0.125–0.25)	0.25 (0.25)
	E-test	0.38 (0.38–0.5)	0.75 (0.75)	1.5 (1–1.5)	0.19 (0.19)	-
Cloxacillin	BMD	0.25 (0.25)	0.25 (0.25)	1 (1)	0.125 (0.125)	0.125 (0.125)
	E-test	0.38 (0.25–0.38)	0.5 (0.38–0.5)	0.75 (0.5–0.75)	0.125 (0.125)	-

## Data Availability

Sequencing information for this study was submitted to the NCBI GenBank database under the BioProject number PRJNA977865.

## Supplementary Materials

The following supporting information can be downloaded at: https://www.mdpi.com/article/10.3390/antibiotics14050449/s1, Table S1: Antimicrobial susceptibility testing of clinical *S. aureus* isolates using MIC strips. Table S2: Antimicrobial susceptibility testing of clinical *S. aureus* isolates using disk diffusion. Table S3: Antimicrobial susceptibility of *E. coli* MG1655 (λDE3) carrying s blaZ variants (A, B, C, D, F). Table S4: Antimicrobial susceptibility of *E. coli* (DH5α) carrying blaIRZ gene cassette variants. Table S5: Description of BlaZ variant F found in the NCBI database. Table S6: List of all MSSA isolates used in this study. Table S7: List of all primers used in this study. Figure S1: Multiple alignment of all BlaZ variants A, B, C, D and F from clinical *S. aureus* bacteraemia isolates and BlaZ from ATCC29213. Figure S2: Multiple alignment of BlaZ variant F from *S. aureus* found using NCBI BlastP PHI.

## Abbreviations

The following abbreviations are used in this manuscript:

MIC	Minimum inhibitory concentration
BORSA	Borderline oxacillin-resistant *Staphylococcus aureus*
MSSA	Methicillin-susceptible *Staphylococcus aureus*
MRSA	Methicillin-resistant *Staphylococcus aureus*
AST	Antibiotic susceptibility testing

## References

[1] McNeil J.C., Sommer L.M., Vallejo J.G., Boyle M., Hulten K.G., Kaplan S.L., Fritz S.A. (2023). Going Back in Time: Increasing Penicillin Susceptibility among Methicillin-Susceptible *Staphylococcus aureus* Osteoarticular Infections in Children. Antimicrob. Agents Chemother..

[2] Rowland S.J., Dyke K.G. (1990). Tn552, a novel transposable element from *Staphylococcus aureus*. Mol. Microbiol..

[3] Novick R.P., Richmond M.H. (1965). Nature and Interactions of the Genetic Elements Governing Penicillinase Synthesis in *Staphylococcus aureus*. J. Bacteriol..

[4] Wang P.Z., Projan S.J., Novick R.P. (1991). Nucleotide sequence of beta-lactamase regulatory genes from staphylococcal plasmid pI258. Nucleic Acids Res..

[5] Bush K., Jacoby G.A. (2010). Updated functional classification of beta-lactamases. Antimicrob. Agents Chemother..

[6] Ambler R.P., Coulson A.F., Frere J.M., Ghuysen J.M., Joris B., Forsman M., Levesque R.C., Tiraby G., Waley S.G. (1991). A standard numbering scheme for the class A beta-lactamases. Biochem. J..

[7] Voladri R.K., Kernodle D.S. (1998). Characterization of a chromosomal gene encoding type B beta-lactamase in phage group II isolates of *Staphylococcus aureus*. Antimicrob. Agents Chemother..

[8] Voladri R.K., Tummuru M.K., Kernodle D.S. (1996). Structure-function relationships among wild-type variants of *Staphylococcus aureus* beta-lactamase: Importance of amino acids 128 and 216. J. Bacteriol..

[9] Hryniewicz M.M., Garbacz K. (2017). Borderline oxacillin-resistant *Staphylococcus aureus* (BORSA)—A more common problem than expected?. J. Med. Microbiol..

[10] McDougal L.K., Thornsberry C. (1986). The role of beta-lactamase in staphylococcal resistance to penicillinase-resistant penicillins and cephalosporins. J. Clin. Microbiol..

[11] Liu H., Buescher G., Lewis N., Snyder S., Jungkind D. (1990). Detection of borderline oxacillin-resistant *Staphylococcus aureus* and differentiation from methicillin-resistant strains. Eur. J. Clin. Microbiol. Infect. Dis..

[12] Chatterjee S.S., Chen L., Gilbert A., da Costa T.M., Nair V., Datta S.K., Kreiswirth B.N., Chambers H.F. (2017). PBP4 Mediates beta-Lactam Resistance by Altered Function. Antimicrob. Agents Chemother..

[13] Hackbarth C.J., Kocagoz T., Kocagoz S., Chambers H.F. (1995). Point mutations in *Staphylococcus aureus* PBP 2 gene affect penicillin-binding kinetics and are associated with resistance. Antimicrob. Agents Chemother..

[14] Nadarajah J., Lee M.J.S., Louie L., Jacob L., Simor A.E., Louie M., McGavin M.J. (2006). Identification of different clonal complexes and diverse amino acid substitutions in penicillin-binding protein 2 (PBP2) associated with borderline oxacillin resistance in Canadian *Staphylococcus aureus* isolates. J. Med. Microbiol..

[15] Tomasz A., Drugeon H.B., de Lencastre H.M., Jabes D., McDougall L., Bille J. (1989). New mechanism for methicillin resistance in *Staphylococcus aureus*: Clinical isolates that lack the PBP 2a gene and contain normal penicillin-binding proteins with modified penicillin-binding capacity. Antimicrob. Agents Chemother..

[16] Griffiths J.M., O’Neill A.J. (2012). Loss of function of the gdpP protein leads to joint beta-lactam/glycopeptide tolerance in *Staphylococcus aureus*. Antimicrob. Agents Chemother..

[17] Corrigan R.M., Abbott J.C., Burhenne H., Kaever V., Grundling A. (2011). c-di-AMP is a new second messenger in *Staphylococcus aureus* with a role in controlling cell size and envelope stress. PLoS Pathog..

[18] Papanicolas L.E., Bell J.M., Bastian I. (2014). Performance of phenotypic tests for detection of penicillinase in *Staphylococcus aureus* isolates from Australia. J. Clin. Microbiol..

[19] Sakoulas G., Nizet V. (2024). Measuring beta-lactam minimum inhibitory concentrations in *Staphylococcus aureus* in the clinical microbiology laboratory: Pinning the tail on the donkey. J. Clin. Microbiol..

[20] Nannini E.C., Stryjewski M.E., Singh K.V., Bourgogne A., Rude T.H., Corey G.R., Fowler V.G., Murray B.E. (2009). Inoculum effect with cefazolin among clinical isolates of methicillin-susceptible *Staphylococcus aureus*: Frequency and possible cause of cefazolin treatment failure. Antimicrob. Agents Chemother..

[21] Richter S.S., Doern G.V., Heilmann K.P., Miner S., Tendolkar S., Riahi F., Diekema D.J. (2016). Detection and Prevalence of Penicillin-Susceptible *Staphylococcus aureus* in the United States in 2013. J. Clin. Microbiol..

[22] Skov R., Lonsway D.R., Larsen J., Larsen A.R., Samulioniene J., Limbago B.M. (2021). Evaluation of methods for detection of beta-lactamase production in MSSA. J. Antimicrob. Chemother..

[23] Ba X., Harrison E.M., Lovering A.L., Gleadall N., Zadoks R., Parkhill J., Peacock S.J., Holden M.T., Paterson G.K., Holmes M.A. (2015). Old Drugs To Treat Resistant Bugs: Methicillin-Resistant *Staphylococcus aureus* Isolates with mecC Are Susceptible to a Combination of Penicillin and Clavulanic Acid. Antimicrob. Agents Chemother..

[24] Baba T., Takeuchi F., Kuroda M., Yuzawa H., Aoki K., Oguchi A., Nagai Y., Iwama N., Asano K., Naimi T. (2002). Genome and virulence determinants of high virulence community-acquired MRSA. Lancet.

[25] Brzoska A.J., Firth N. (2013). Two-plasmid vector system for independently controlled expression of green and red fluorescent fusion proteins in *Staphylococcus aureus*. Appl. Environ. Microbiol..

[26] Baba T., Bae T., Schneewind O., Takeuchi F., Hiramatsu K. (2008). Genome sequence of *Staphylococcus aureus* strain Newman and comparative analysis of staphylococcal genomes: Polymorphism and evolution of two major pathogenicity islands. J. Bacteriol..

[27] Livorsi D.J., Crispell E., Satola S.W., Burd E.M., Jerris R., Wang Y.F., Farley M.M. (2012). Prevalence of blaZ gene types and the inoculum effect with cefazolin among bloodstream isolates of methicillin-susceptible *Staphylococcus aureus*. Antimicrob. Agents Chemother..

[28] Banerjee S., Pieper U., Kapadia G., Pannell L.K., Herzberg O. (1998). Role of the omega-loop in the activity, substrate specificity, and structure of class A beta-lactamase. Biochemistry.

[29] Konstantinovski M.M., Veldkamp K.E., Lavrijsen A.P.M., Bosch T., Kraakman M.E.M., Nooij S., Claas E.C.J., Gooskens J. (2021). Hospital transmission of borderline oxacillin-resistant *Staphylococcus aureus* evaluated by whole-genome sequencing. J. Med. Microbiol..

[30] Monk I.R., Tree J.J., Howden B.P., Stinear T.P., Foster T.J. (2015). Complete Bypass of Restriction Systems for Major *Staphylococcus aureus* Lineages. mBio.

[31] O’Callaghan C.H., Morris A., Kirby S.M., Shingler A.H. (1972). Novel method for detection of beta-lactamases by using a chromogenic cephalosporin substrate. Antimicrob. Agents Chemother..

