# Supplementary material for: Genetic Variation in the blaZ Gene Leading to the BORSA Phenotype in Staphylococcus aureus
Source: Antibiotics (Basel). 2025 Apr 29;14(5):449. doi: 10.3390/antibiotics14050449 (PMC12108325; doi:10.3390/antibiotics14050449)
Supplement: Supplementary file 1 [file antibiotics-14-00449-s001.zip › Supplemental files.pdf]

**Table S1. Antimicrobial susceptibility testing of clinical *Staphylococcus aureus* isolates using MIC gradient strips**. Values displayed are the MIC median (mg/L) and ranges for variant A, B and C (n = 10), variant D (n = 8) and variant F (n= 3).

| Antibiotic | *blaZ* variants | | | | | Controls | |
|---|---|---|---|---|---|---|---|
| | A | B | C | D | F | S | ATCC29213 |
| Cefuroxime | **0.875** (0.38-1) | **1** (0.5-2) | **1** (0.75-1.5) | **1** (0.75-1.5) | **1.5** (1-1.5) | **0.75** (0.75) | **0.75** (0.75) |
| Ampicillin-sulbactam | **0.125** (0.064-0.38) | **0.38** (0.125-0.75) | **0.25** (0.19-0.5) | **0.25** (0.064-0.38) | **0.38** (0.19-0.38) | **0.047** (0.047) | **0.125** (0.125) |
| Amoxicillin-clavulanic acid | **0.11** (0.023-0.19) | **0.19** (0.125-0.75) | **0.22** (0.125-0.38) | **0.125** (0.094-0.25) | **0.25** (0.094-0.5) | **0.064** (0.064) | **0.125** (0.125) |

**Table S2. Antimicrobial susceptibility testing of clinical *Staphylococcus aureus* isolates using disk diffusion**. Values displayed are the median zone of inhibition (mm) and ranges for variant A, B and C (n = 10), variant D (n = 8) and variant F (n= 3)

| Antibiotic | *blaZ* variants | | | | | Controls | |
|---|---|---|---|---|---|---|---|
| | A | B | C | D | F | S | ATCC29213 |
| Benzylpenicillin | **14** (8-18) | **10** (6-14) | **8** (6-11) | **12** (6-22) | **6** (6) | **32** (25-37) | **14** (14-15) |
| Ampicillin | **17** (13-21) | **13** (9-17) | **11** (6-14) | **15** (12-26) | **8** (6-11) | **30** (24-35) | **14** (13-16) |
| Piperacillin | **21.5** (19-24) | **19** (17-22) | **16** (15-19.5) | **19** (17-24) | **12** (9-14) | **34** (30-38) | **19** (19) |
| Oxacillin | **22** (18-26) | **19** (14-22) | **18** (14-20) | **21** (18-24) | **8.5** (6-11) | **24** (20-29) | **23** (22.5-23) |
| Cefoxitin | **31** (29-33) | **29** (27-34) | **29** (26-32) | **31** (29-33) | **29** (29-30) | **30.5** (29-34) | **27** (26.5-28) |
| Cefuroxime | **34** (29-37) | **31.5** (30-33) | **31** (28-37) | **32** (26-35) | **23** (22-24) | **32.5** (30-34) | **30.5** (30-30.5) |
| Mecillinam | **6** (6) | **6** (6) | **6** (6) | **6** (6) | **6** (6) | **6** (6-12) | **6** (6) |
| Meropenem | **42** (38-44) | **39** (37-40) | **38** (35-42) | **40** (38-41) | **33.5** (32-37) | **41** (39-45) | **37** (36.5-37) |
| Ampicillin-sulbactam | **28** (24-32) | **22** (19-24) | **19.5** (18-21) | **24.5** (21-33) | **22.5** (22-23) | **39.5** (37-43) | **25.5** (25-26) |
| Amoxicillin-clavulanic acid | **32.5** (28-37) | **24.5** (22-29) | **24.5** (22-28) | **29.5** (26-39) | **26** (24-28) | **39** (37-43) | **32.5** (31.5-33) |
| Piperacillin-tazobactam | **25.5** (21-30) | **20** (17-23) | **19.5** (18-23) | **24.5** (21-34) | **22** (21-23) | **35** (33-39) | **26** (25-27) |

**Table S3. Antimicrobial susceptibility of *E. coli* MG1655 (λDE3) carrying *blaZ* variants (A, B, C, D, F).** MICs (mg/L) were determined using broth microdilution. Values displayed are the MIC (mg/L) median and ranges (n = 3).

| **Antibiotics** | ***blaZ* variants** | | | | | **Controls** | |
|---|---|---|---|---|---|---|---|
| | **A** | **B** | **C** | **D** | **F** | **pET26B** | **ATCC29213** |
| Benzylpenicillin | **128** (64-128) | **32** (32-64) | **256** (256) | **128** (128) | **128** (128) | **16** (16) | **128** (128-256) |
| Ampicillin | **64** (16-64) | **32** (16-32) | **64** (64) | **32** (32-64) | **512** (128-512) | **<16** (<16) | **128** (64-128) |
| Oxacillin | **256** (256) | **256** (256-512) | **256** (256) | **256** (256) | **256** (256) | **256** (256) | **256** (256-512) |
| Cloxacillin | **256** (256-512) | **512** (512) | **256** (256-512) | **256** (256-512) | **512** (512) | **256** (256-512) | **256** (256-512) |
| Cefuroxime | **2** (2) | **4** (2-4) | **2** (2) | **2** (2) | **4** (2-4) | **2** (2) | **2** (2-4) |

**Table S4. Antimicrobial susceptibility of *E. coli* (DH5α) carrying *blaIRZ* gene cassette variants.**
*blaIRZ* gene cassette from variant A, variant A carrying mutation S216T and variant F are carried on pBL_A, pBL_AF and pBL_F respectively. pBL_N backbone vector that does not carry *blaIRZ* gene cassette was used as control .
MICs (mg/L) were determined using broth microdilution in LB medium. Values displayed are the median and ranges (n = 3).

| **Antibiotics** | ***blaZ* variants** | | | **Controls** |
|---|---|---|---|---|
| | **A** | **AF** | **F** | **pBL_N** |
| Ampicillin | **16** (16) | **128** (64-128) | **>512** (>512) | **4** (4) |

**Table S5. Description of BlaZ variant F found in NCBI database.**

| ID | Organism | Isolation Source | Isolate/strain | Host | Geoc_locname |
|---|---|---|---|---|---|
| HCS9142876.1 | *S. aureus* | | AUSMDU00023298 | Homo sapiens | Australia: Victoria |
| HCZ9853562.1 | *S. aureus* | Skin and soft tissue infection | M8420 | Homo sapiens | Denmark: Copenhagen |
| HDJ6869879.1 | *S. aureus* | SSTI | M5300 | Homo sapiens | Denmark: Copenhagen |
| HGX3308986.1 | *S. aureus* | Pus/Wound swab | FIJ0611 | Homo sapiens | Fiji: Suva |
| HCY5423107.1 | *S. aureus* | Right-Cubital-Fossa | 050-CD1 | Homo sapiens | Mexico: Mexico City |
| HCY5423663.1 | *S. aureus* | Right-Cubital-Fossa | 050-CD1 | Homo sapiens | Mexico: Mexico City |
| HCZ6912216.1 | *S. aureus* | Right-Cubital-Fossa | 071-CI1 | Homo sapiens | Mexico: Mexico City |
| HCZ6912305.1 | *S. aureus* | Right-Cubital-Fossa | 071-CI1 | Homo sapiens | Mexico: Mexico City |
| HCZ6912311.1 | *S. aureus* | Right-Cubital-Fossa | 071-CI1 | Homo sapiens | Mexico: Mexico City |
| HCZ6923041.1 | *S. aureus* | Right-Cubital-Fossa | 071-CI5 | Homo sapiens | Mexico: Mexico City |
| HDF6520505.1 | *S. aureus* | | RIVM_M088715 | Homo sapiens | Netherlands |
| HFO0490716.1 | *S. aureus* | Bacterial Isolate from aseptic quarter milk sample | K593 | Bos taurus | New Zealand: Southland |
| HDA1970352.1 | *S. aureus* | clinical sample | H120420116-223-A0-2 | Homo sapiens | United Kingdom: London |
| HDA1970356.1 | *S. aureus* | clinical sample | H120420116-223-A0-2 | Homo sapiens | United Kingdom: London |
| MDU7274752.1 | *S. aureus* | infant feces | | | USA: Pittsburgh |
| HDD7858638.1 | *S. aureus* | | MPROS0043 | | |

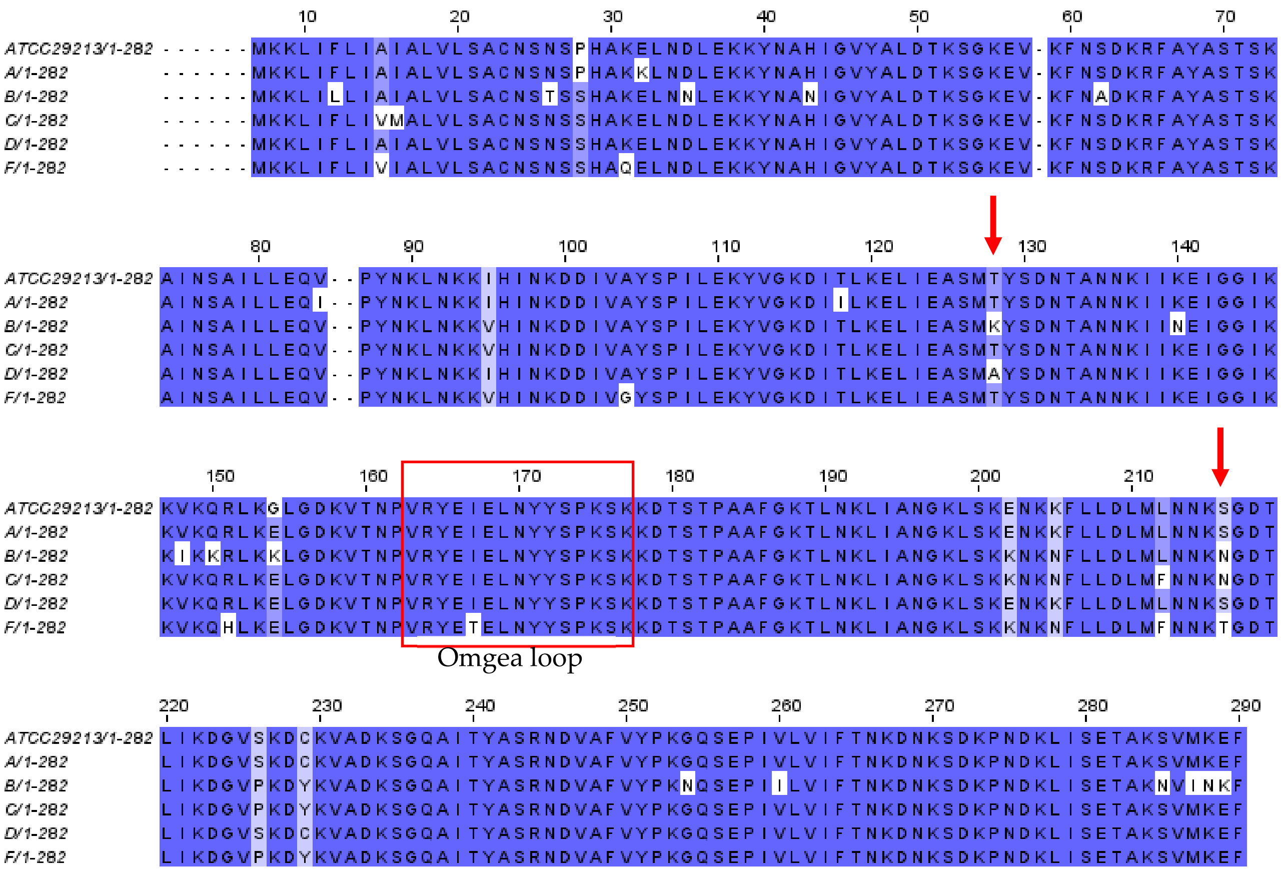


<u>Figure S1:</u> **Multiple alignment of all BlaZ variants A, B, C, D, F from clinical *Staphylococcus aureus* bacteraemia isolates and BlaZ from ATCC29213.** The amino acid numbering is according to Ambler's standard numbering scheme, which includes the gaps on position 58, 85 and 86. Position 128 and 216 (indicated with a red arrow on the figure) determine the variant (A, B, C, D, F) of the specific BlaZ protein. BlaZ$_{ATCC29213}$ gives rise to a BlaZ variant A.

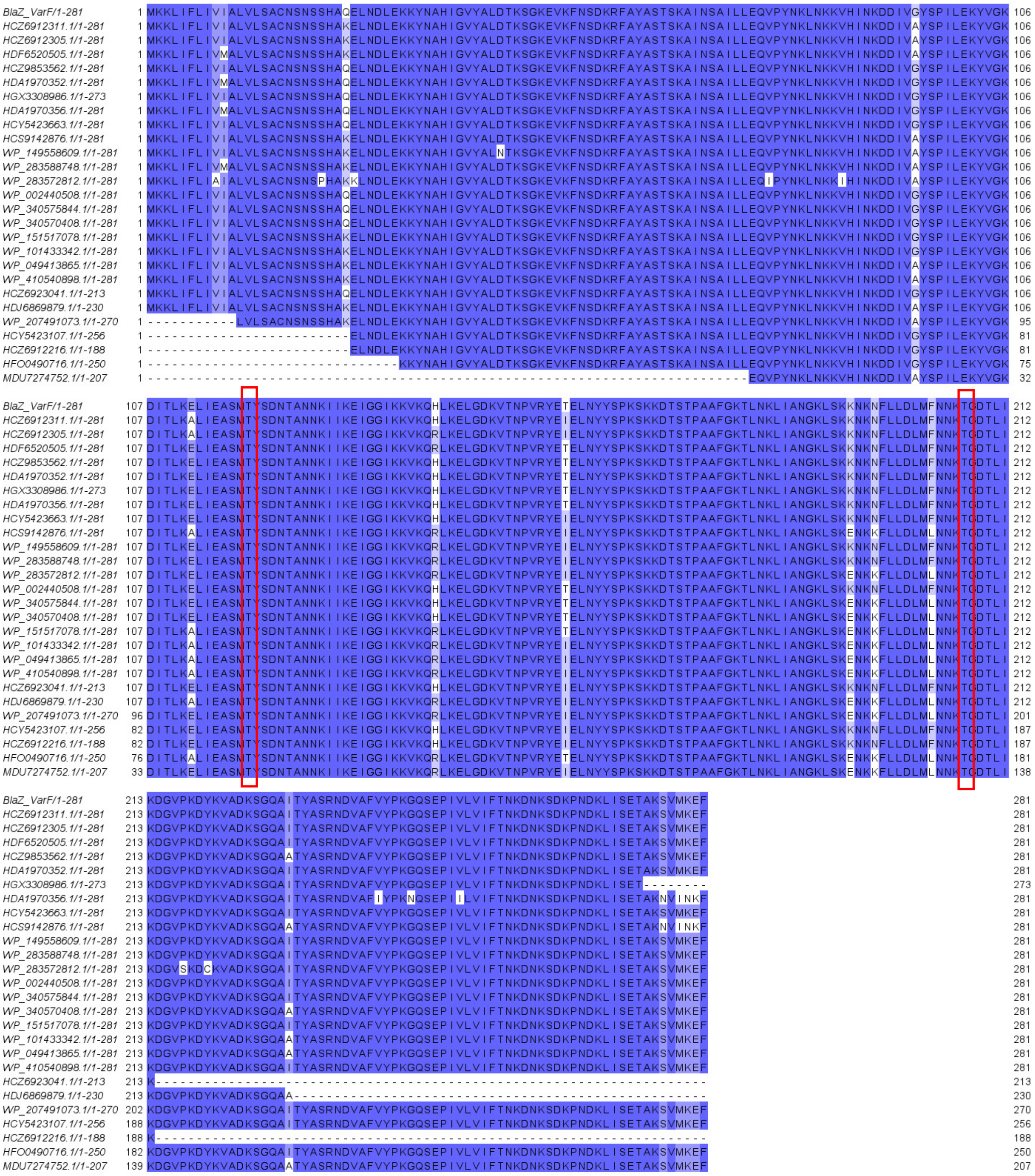

**<u>Figure S2:</u> Multiple alignment of BlaZ variants F from *Staphylococcus aureus* found using NCBI BlastP PHI.** Shown alignment of the best hit using BlastP PHI using using full protein sequence of variant F and PHI pattern=TXXXXXXXXXXXXXXXXXXXXXXXXXXXXXXXXXXXXXXXXXXXXXXXXXXXXXXXXXXXXXXXXXXXXXXXXX XXXXXXXXXXXXXXT. Position 128 and 216 (Ambler) are highlighted.
